# Cochlear Amplification Modulates Synaptic Transmission at the Endbulb of Held Synapse in the Cochlear Nucleus

**DOI:** 10.1523/JNEUROSCI.1673-25.2026

**Published:** 2026-02-17

**Authors:** Fang Wang, Yige Li, Geng-Lin Li

**Affiliations:** ^1^Department of Otorhinolaryngology, ENT institute, and NHC Key Laboratory of Hearing Medicine, Eye & ENT Hospital, Fudan University, Shanghai, 200031, China; ^2^Institutes of Brain Science, State Key Laboratory of Medical Neurobiology, and MOE Frontiers Center for Brain Science, Fudan University, Shanghai, 200031, China

**Keywords:** bushy cell, cochlear amplification, inner hair cell, paired-pulse plasticity, prestin, readily releasable pool of synaptic vesicles

## Abstract

In the mammalian cochlea upon acoustic stimulation, outer hair cells (OHCs) push and pull the basilar membrane, amplifying its vibration and therefore expanding the dynamic range of hearing. As a result, spiking patterns in auditory nerve fibers (ANFs) are believed to be significantly different, but how the central nervous system adapts to this substantial change is poorly understood. In this study, we took advantage of *Prestin^−/−^* mice of either sex where prestin, the motor protein in OHCs, was genetically knocked out, therefore removing cochlear amplification completely without changing the cellular structure of the cochlea significantly. While exocytosis from inner hair cells in the cochlea was largely intact, transmission at the endbulb of Held synapse between ANFs and bushy cells in the cochlear nucleus was significantly changed in *Prestin^−/−^* mice. Specifically, excitability of bushy cells was significantly increased, due to combination of slightly more depolarized resting membrane potential, increased membrane input resistance, and smaller and briefer afterhyperpolarization. Furthermore, synaptic strength was greatly reduced, caused by substantial decrease in the readily releasable pool (RRP) of synaptic vesicles. Significantly, paired-pulse plasticity at this synapse was reversed from depression in WT mice to facilitation in *Prestin^−/−^* mice, likely caused by quicker refilling of RRP observed in *Prestin^−/−^* mice. In conclusion, we found that transmission at the endbulb of Held synapse is significantly altered in absence of cochlear amplification, revealing interplay between the peripheral and central processing of auditory signals that contributes to expanded dynamic range of hearing seen in mammals and humans.

## Significance Statement

The mammalian cochlea is an amazing sensory apparatus with remarkable sensitivity, largely owning to cochlear amplification that is estimated to be ∼1,000 folds. As a result, auditory nerve fibers are expected to fire spikes with significantly different patterns. If and how central circuits adapt to this substantial change of cochlear input are poorly understood. To address this fundamental question, we abolished cochlear amplification in *Prestin^−/−^* mice and examined functional changes in the cochlear nucleus. We found that excitability of bushy cells was increased, and transmission at the endbulb of Held synapse was significantly altered. We therefore revealed an active interaction between the hearing organ and central circuits that expands our understanding of hearing in general.

## Introduction

Although all vertebrates have hair cells in their vestibular and hearing organs to detect motion and vibration, only the emergence of inner and outer hair cells (IHCs and OHCs) in the cochlea of mammals allows them to hear sound of a much broader frequency and dynamic range (Lipovsek and Elgoyhen, 2023). In the cochlea, IHCs and OHCs are arranged in parallel between the tectorial and basilar membrane, so that their hair bundles are activated simultaneously in synchronization, converting mechanical vibration into changes in graded membrane potential (Fettiplace and Hackney, 2006). Prestin, a unique motor protein, is densely expressed in the lateral wall of plasma membrane in OHCs, allowing them to contract upon depolarization and elongate upon hyperpolarization (Liberman et al., 2002). With this electromotility, OHCs are capable of pushing and pulling the basilar membrane, precisely phase-locked to acoustic cycles in real time, amplifying its vibration mechanically by ∼1,000 folds (Davis, 1983; Ashmore, 2008). This cochlear amplification allows mammals and humans to hear sound as weak as ∼20 μPa (or 0 dB SPL), well beyond 10 kHz, and up to 150 kHz in some species (Johnson, 2011; Lipovsek and Elgoyhen, 2023).

With cochlear amplification, temporal structures of spiking in auditory nerve fibers (ANFs) can be dramatically different. Sound-evoked spikes in mammalian ANFs are known to be sharply tuned in frequency, but when OHCs in the cochlea are chemically ablated, the frequency tuning in ANFs is severely reduced, with nearly half of them becoming broadly tuned in frequency (Dallos and Harris, 1978; Liberman and Dodds, 1984; Kiang et al., 1986). On the one hand, cochlear amplification is required to drive most sensitive ANFs to respond very weak sounds. On the other hand, cochlear amplification makes ANFs more selective, preventing them from being recruited in response to sounds of other than their characteristic frequency. Finally, in response to pure tone stimulation, mammalian ANFs fire spikes that are phase-locked to the acoustic cycle, and this phase-locking of spikes is significantly degraded when cochlear OHCs are chemically ablated (Woolf et al., 1981).

But if and how central circuits adapt to this change of spiking in ANFs brought by cochlear amplification are poorly understood. Throughout the central nervous system, neuronal excitability and synaptic strength are delicately and dynamically modulated by neural inputs (Zhang and Linden, 2003; Magee and Grienberger, 2020). In whole animals, loss of cochlear amplification has been shown to reduce temporal processing efficacy in neurons in the inferior colliculus (Walton et al., 2018). However, it remains unclear if and how this change is caused by changes within the inferior colliculus or by changes in central circuits of more upstream.

To address this fundamental question, we decided to focus on the endbulb of Held synapse, the first central synapse in the auditory pathways, formed between ANFs and bushy cells in the cochlear nucleus in the brainstem. It has been shown that this synapse is dynamically and reversibly modulated by changes in cochlear input (Wright et al., 2014; Ngodup et al., 2015; Zhuang et al., 2017). To remove cochlear amplification, we took advantage of a *Prestin^−/−^* mouse line where prestin is genetically knocked out (Zhang et al., 2018). We performed patch-clamp recording in vitro in hair cells in whole-mount cochleae, and in bushy cells in brainstem slices, prepared from both WT and *Prestin^−/−^* mice. We found that while exocytosis from IHCs in the cochlea was largely intact, excitability of bushy cells was significantly increased, and transmission at the endbulb of Held synapse was greatly altered, in *Prestin^−/−^* mice lacking cochlear amplification. We conclude that this very first central synapse in the auditory pathways is actively modulated by cochlear amplification in the periphery.

## Materials and Methods

### Electrophysiology in vivo

The *Prestin* knock-out mouse line (*Prestin^−/−^*) was generated by Dr. Zhiyong Liu in a published study (Zhang et al., 2018) and kindly provided to us as a gift. The mice were bred in a centralized animal facility in Fudan University, and protocols regarding animal housing and handling are consistent with the Guide for the Care and Use of Laboratory Animals (National Institutes of Health), reviewed, and approved by the Animal Care and Use Committee of Fudan University. Wild-type (WT) and *Prestin^−/−^* mice of either sex with an age range of P18-30 were used. Auditory brainstem responses (ABRs) and distortion product otoacoustic emissions (DPOAEs) were recorded in a soundproof chamber with a RZ6 acquisition system (Tucker-Davis Technologies). Briefly, the mice were anesthetized with dexmedetomidine hydrochloride (450 μl/kg) and zoletil 50 (45 mg/kg) through intraperitoneal injection. For ABRs, three subdermal needle electrodes were inserted at the left rump, the mastoid region of the right ear and the vertex, for ground, reference, and recording, respectively (Song et al., 2008). Clicks or pure tone pips of 4, 5.6, 8, 11.3, 16, 22.6, and 32 kHz were delivered via a speaker that was placed 10 cm away from the outer ear, from a maximum sound intensity of 90 dB SPL, with a decrement of 5 dB successively to 20 dB SPL. For the same sound stimulus, a total of 512 responses were collected and averaged, and the ABR threshold was defined as the lowest sound intensity that evoked a visible and reproducible waveform. For DPOAEs, two simultaneous pure tones with a frequency ratio (ƒ_2_/ƒ_1_) of 1.2 and equal sound intensity were delivered closed-field to the external acoustic meatus continuously and DPOAEs were recorded (Kemp and Brown, 1983). The sound stimuli were presented at a center frequency of 5.6, 8, 11.3, 16, 22.6, and 32 kHz from a maximum sound intensity of 80 dB SPL with a decrement of 5 dB successively to 20 dB SPL. DPOAE at the frequency 2ƒ_1_−ƒ_2_ was analyzed and DPOAE thresholds were defined as the lowest sound intensity that generated a DPOAE with amplitude of 3 dB higher than the noise floor.

### Patch-clamp recording in whole-mount cochleae

The mice were anesthetized, the cochlea was isolated, and the apical turn of the basilar membrane was excised in one of the two extracellular solutions (see below). The tissue was visualized under a 60× water-immersion lens, and IHCs and OHCs were identified through their location and specific arrangement of stereocilia. Patch-clamp recordings were made with a Heka patch-clamp amplifier (EPC10/2 USB, HEKA), driven by a PC computer running PatchMaster (Heka).

For recording in IHCs, the extracellular solution contained the following (in mM): 123 NaCl, 5.8 KCl, 5 CaCl_2_, 0.9 MgCl_2_, 10 HEPES, 10 d-glucose, 0.7 NaH_2_PO_4_, and 2 sodium pyruvate, pH adjusted to 7.40, osmolarity to 300 mmol/kg, bubbled with pure O_2_ for 10 min before use. To isolate Ca^2+^ current in IHCs, we used a Cs^+^-based intracellular solution containing the following (in mM): 135 Cs-methanesulfonate, 10 CsCl, 10 HEPES, 10 TEA-Cl, 2 EGTA, 3 Mg-ATP, and 0.5 Na-GTP (pH 7.2, 290 mmol/kg). The liquid junction potential was measured and approximated to be 20 mV, which was subtracted offline. The cell was held under voltage clamp at −100 mV between stimulations. For recording of Ca^2+^ current, a voltage ramp from −100 to 60 mV in 300 ms was applied. To remove the leak current in Ca^2+^ currents, we first fitted the initial part of the current response (50 ms) to a straight line, obtained the slope as the leak conductance, extrapolated the line with the slope to estimate the leak current for the rest of the voltage range, and then subtracted it from the original current recording. For capacitance measurement, exocytosis of synaptic vesicles was induced by a voltage step protocol, where the cell was depolarized to −20 mV for a varied duration from 10 to 500 ms. Meanwhile, sinewaves of 50 mV (peak-to-peak) and 1 kHz were superposed on the holding potential before and after the stimulation, and the resulting current responses were used to calculate whole-cell capacitance through the “LockIn” feature and the “Sine + DC” method. Capacitance changes (Δ*C_m_*) were calculated for individual IHCs and plotted against the stimulation time (*t*), which were fitted to a combination of an exponential function for releasing of readily releasable pool (RRP) of synaptic vesicles (*C_m_*_,RRP_, τ_RRP_), a straight line for sustained release of synaptic vesicles (*R*_sustained_), and a constant for capacitance drift (*C_m_*_,drift_):

$$\Delta C_{m}\;\left(t\right)=C_{m,\mathrm{RRP}}\;\cdot \;\left(1\;-\;\exp\;\left(\frac{t}{\tau_{\mathrm{RRP}}}\right)\right)\;+\;R_{\mathrm{sustained}}\;\cdot \;t\;+\;C_{m,\mathrm{drift}}.$$

After *C_m_*_,RRP_ and *R*_sustained_ were extracted from curve fitting, the values in capacitance were converted to numbers of synaptic vesicles (Fig. 3*E*,*F*) with a ratio of 37 aF per synaptic vesicle (Lenzi et al., 1999).

For recording in OHCs, the extracellular solution contained (in mM) 136 NaCl, 1 CaCl_2_, 1 MgCl_2_, 10 HEPES, and 10 d-glucose (pH 7.25, 300 mmol/kg), and the intracellular solution contained (in mM) 136 NaCl, 1 CaCl_2_, 1 MgCl_2_, 10 HEPES, and 10 EGTA (pH 7.25, 300 mmol/kg). No K^+^ was included in either side of the solution to maximally block different types of K^+^ currents in OHCs, allowing capacitive current for charging and discharging the membrane to be isolated and used to probe changes in nonlinear capacitance, a measure of prestin function in OHCs. Symmetric ionic composition was used to eliminate the liquid junction potential. Indeed, the liquid junction potential was measured to be ∼0 mV, so no correction is needed. For measurement of nonlinear capacitance, the cell was subjected to a voltage protocol by turning on the “Ext. Stim. Input” feature in the amplifier window in the PatchMaster software and combining a sinewave of 1 kHz and 20 mV (peak-to-peak) and a voltage ramp from −160 to 160 mV, the latter of which was generated through a DA channel in the amplifier and fed to the external stimulation connection through a BNC cable.

### Patch-clamp recording in brainstem slices

The animal was anesthetized and decapitated, and coronal or sagittal brainstem slices of 200−250 μm thick were prepared with a vibratome (Leica, VT1200 S) in sucrose-based, ice-cold cutting solution containing the following (in mM): 230 sucrose, 10 d-glucose, 25 NaHCO_3_, 3 MgCl_2_, 0.1 CaCl_2_, 2.5 KCl, 1.25 NaH_2_PO_4_, 0.4 sodium ʟ-ascorbate, 2 sodium pyruvate, and 3 myo-inositol, bubbled continuously with 95% O_2_ and 5% CO_2_. Brainstem slices with the anteroventral cochlear nucleus were collected and incubated at 34°C for at least 30 min in the artificial cerebral spinal fluid (ACSF) containing the following (in mM): 125 NaCl, 10 d-glucose, 25 NaHCO_3_, 1.8 MgCl_2_, 1.2 CaCl_2_, 2.5 KCl, 1.25 NaH_2_PO_4_, 0.4 sodium ʟ-ascorbate, 2 sodium pyruvate, and 3 myo-inositol, bubbled continuously with 95% O_2_ and 5% CO_2_. The brainstem slice was then transferred to a recording chamber with recirculating ACSF at room temperature. For voltage-clamp recording, the intracellular solution contained the following (in mM): 130 Cs-methanesulfonate, 10 CsCl, 10 HEPES, 5 EGTA, 4 Mg-ATP and 0.3 Na_2_-GTP, 10 Na_2_-phosphcreatine and 3 QX314-Cl (pH 7.3, 290 mOsm). In addition, strychnine of 2 μM was added into ACSF to block inhibition. For current-clamp recording, the intracellular solution contained the following (in mM): 130 K-gluconate, 10 KCl, 10 HEPES, 2 EGTA, 10 creatine phosphate, 3 Mg-ATP, and 0.5 Na_2_-GTP (pH 7.3, 290 mOsm). The liquid junction potential was measured and approximated to be 20 mV and subtracted offline.

Bushy cells were identified under current clamp by their characteristic firing of one to a few spikes in response to prolonged current injections (Wu and Oertel, 1984; Xie, 2016; Antunes et al., 2020) and under voltage-clamp by their large amplitude (>1 nA) and all-or-none synaptic currents. In mice, spherical and globular BCs are not distinct regionally or physiologically; therefore, all the recordings were pooled together (Ferragamo and Oertel, 2001; Lauer et al., 2013). Series resistance was typically 5–15 MΩ, uncompensated. The resting membrane potential was measured by holding the cell at zero current under current clamp (Fig. 6*B*). To probe excitability, a current step protocol was applied under current, by injecting step currents of 500 ms, from −100 to 600 pA, with an increment of 100 pA. Once a range was found where the cell started to fire spikes, step currents with a finer increment of 10 pA were applied, allowing us to determine the current threshold more precisely (Fig. 6*D*). Based on these recordings, the voltage threshold (Fig. 6*E*) was determined as the voltage at which the dV/dt reached 20 V/s in the depolarizing phase, the afterhyperpolarization (AHP) amplitude was calculated as the voltage difference between the voltage threshold and the voltage trough following the spike (Fig. 6*H*), and the AHP time was measured as the time interval in between (Fig. 6*I*).

Evoked EPSCs were elicited by stimulating the auditory nerve with brief voltage pulses of 0.1 ms and 0.1–10 V, generated through a Master-9 Pulse Stimulator (AMPI) and delivered through a suction pipette filled with ACSF. For EPSCs evoked with single stimulus, the quantal content was calculated by dividing the current amplitude with the average amplitude of spontaneous EPSCs (Fig. 8*G*). For EPSCs evoked by 50 stimuli delivered at 100 Hz, the sustained release rate (SRR) was defined as the average quantal content of the last 20 EPSCs divided by stimulation interval (i.e., 10 ms), and the readily releasable pool (RRP) of synaptic vesicles was calculated by subtracting 50 times of the average quantal content of the last 20 EPSCs from the summation of quantal contents for all 50 EPSCs (Fig. 10*C*,*D*).

### Immunohistochemistry

For staining of whole-mount cochleae, the mice were anesthetized and decapitated, and the cochleae were isolated and transferred to 4% paraformaldehyde, kept at 4°C overnight. Then the cochleae were decalcified in EDTA (120 mM) for 3–4 d, and the basilar membranes were dissected and collected. The primary antibodies included anti-prestin (1:200, Santa Cruz Biotechnology), anti-Vglut3 (1:500, Synaptic System), anti-myosin 7a (1:500, Pretuos), and anti-Ctbp2 (1:500, Boster). The secondary antibodies (Alexa Fluor 488, 568, and 647) that were compatible with different combinations of the primary antibodies were selected. Subsequently, the tissue was counterstained with DAPI (1:1,000, Thermo Scientific) or Hoechst (1:1,000, Thermo Scientific) to visualize cellular nuclei.

For staining of brainstem slices, the mice were anesthetized, perfused with 10 ml PBS and 10 ml 4% paraformaldehyde, and then decapitated; the cochlear nucleus was removed entirely and immediately transferred to 4% paraformaldehyde, kept at 4°C overnight. Then the tissue was immersed in 15% sucrose solution for 1 d and in 30% sucrose solution for 2 d for dehydration. After embedded in OCT, cochlear nuclei were sectioned sagittally at a thickness of 40 μm on a sliding microtome. The prepared cryosections were rehydrated in 0.1 M PBS for 40 min and treated with 10% FBS in 1% PBST at 4°C overnight. The sections were then incubated overnight at 4°C with the mouse anti-Neurofilament 200 (1:1,000, Merck). After washing with PBS, they were incubated with the secondary antibody Alexa Fluor 647 goat anti-mouse for 2 h at room temperature and then with green fluorescent Nissl staining (1:500, N-21480, Molecular Probes) for 20 min. Subsequently, the sections were washed with PBS and counterstained with DAPI (1:1,000, Thermo Scientific) to visualize cellular nuclei and finally mounted after washing with PBS. After staining, serial sections of the cochlear nucleus were digitally scanned for analysis. The width of auditory nerve stump in each section was measured individually in ImageJ, and the maximum value among different serial sections was determined as the diameter of auditory nerve. In addition, the magnocellular region of VCN was manually outlined in each serial section, as illustrated in Figure 4*A*, and the area of each section was quantified in ImageJ. The volume of VCN was calculated by summing areas for all sections and multiplying the sum by the section thickness (40 μm).

### Experimental design and statistical analyses

Data were collected in parallel from WT and *Prestin^−/−^* mice and analyzed with BioSigRP (Tucker-Davis Technologies), ImageJ, Igor Pro (WaveMetrics) and GraphPad Prism (GraphPad). For comparisons between two groups, the data were tested first for normality, and then parametric *t* tests were performed for data with normal distribution, while nonparametric Mann–Whitney tests were performed otherwise. For multiple comparisons, two-way analysis of variance was employed, followed by the Sidak post hoc test when necessary. For the cumulative distribution of minimal stimulation presented in Figure 8*D*, the log-rank test was used to assess the significance of difference. In comparison with the value of 1 presented in Figure 9*C*, one-sample *t* test was performed for normally distributed data, while the Wilcoxon signed rank test was performed otherwise. Data are presented as mean ± standard error of the mean, *n* denotes the number of samples, *N* denotes the number of animals, and the statistical significance was defined as *p* < 0.05 for all tests.

## Results

### Inner hair cell ribbon synapses were largely intact in *Prestin^−/−^* mice

**Figure 1.**
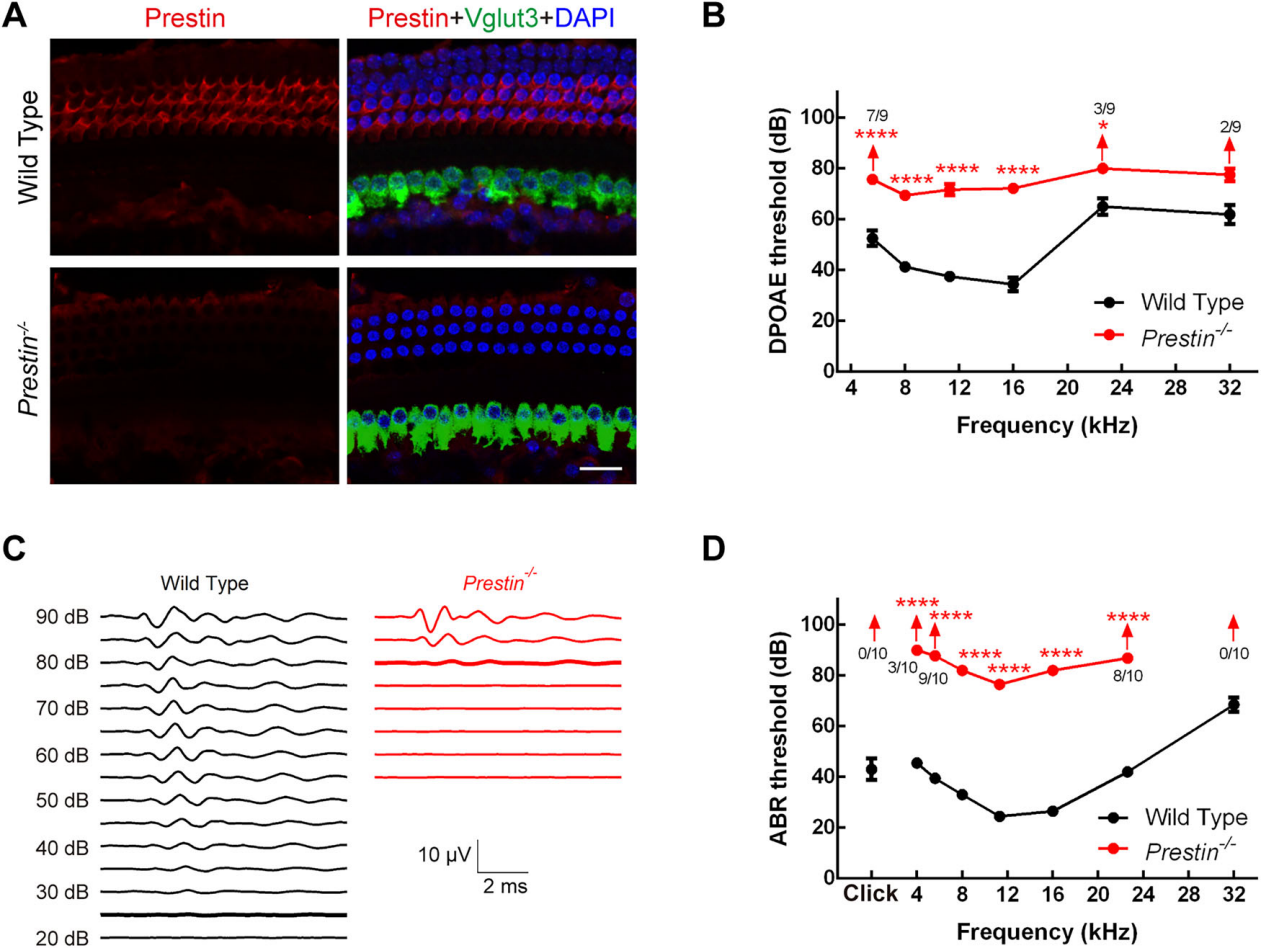
Hearing thresholds were greatly elevated in *Prestin^−/−^* mice. ***A***, Images of whole-mount cochleae triple immunolabeled for prestin, Vglut3, and DAPI in WT and *Prestin^−/−^* mice. While outer hair cells (OHCs) were still in place, prestin expression on their lateral membrane was completely eliminated in *Prestin^−/−^* mice. Scale bar, 20 μm. ***B***, thresholds of DPOAE in WT (*N* = 8 animals) and *Prestin^−/−^* mice (*N* = 9), showing that DPOAE was either undetectable or its thresholds were greatly elevated. ***C***, Representative ABRs to tone bursts of 16 kHz from a pair of WT and *Prestin^−/−^* mice. Responses at thresholds are depicted in bold (25 dB for WT, 80 dB for *Prestin^−/−^*). ***D***, Thresholds of ABRs in WT (*N* = 10) and *Prestin^−/−^* mice (*N* = 10), showing that hearing thresholds in *Prestin^−/−^* mice were greatly elevated for both clicks and pure tones across all frequencies tested. NS means *p* > 0.05. *, **, ***, and **** indicate *p* < 0.05, 0.01, 0.001, and 0.0001, respectively, and up arrow indicates no detectable response.

The *Prestin^−/−^* mice were generated in a previous study (Zhang et al., 2018), and we obtained it from Dr. Zhiyong Liu as a gift. We first performed immunolabeling in whole-mount cochleae of WT and *Prestin^−/−^* mice, and we found that in *Prestin^−/−^* cochleae one row of IHCs was largely intact and that three rows of OHCs were also in place (Fig. 1*A*). As expected, the prestin expression on the lateral plasma membrane of OHCs was completely eliminated in *Prestin^−/−^* cochleae (Fig. 1*A*, bottom). The lack of prestin in OHCs is likely to greatly reduce cochlear amplification, and as a result, the distortion product otoacoustic emission (DPOAE) in *Prestin^−/−^* mice is expected to be reduced as well. Indeed, the DPOAE thresholds in *Prestin^−/−^* mice were increased by 20 dB or more across all frequencies tested (Fig. 1*B*). The removal of the cochlear amplification is also likely to reduce the overall hearing sensitivity. As expected, in a pair of examples, we found that the threshold to 16 kHz pure tone was dramatically increased from 25 dB SPL in the WT mouse to 80 dB SPL in the *Prestin^−/−^* mouse (Fig. 1*C*). Based on the data collected from multiple animals, the thresholds were greatly elevated not only for clicks but also for pure tones of all frequencies tested (Fig. 1*D*). Taken together, these results suggest that cochlear amplification is successfully removed in these *Prestin^−/−^* mice.

**Figure 2.**
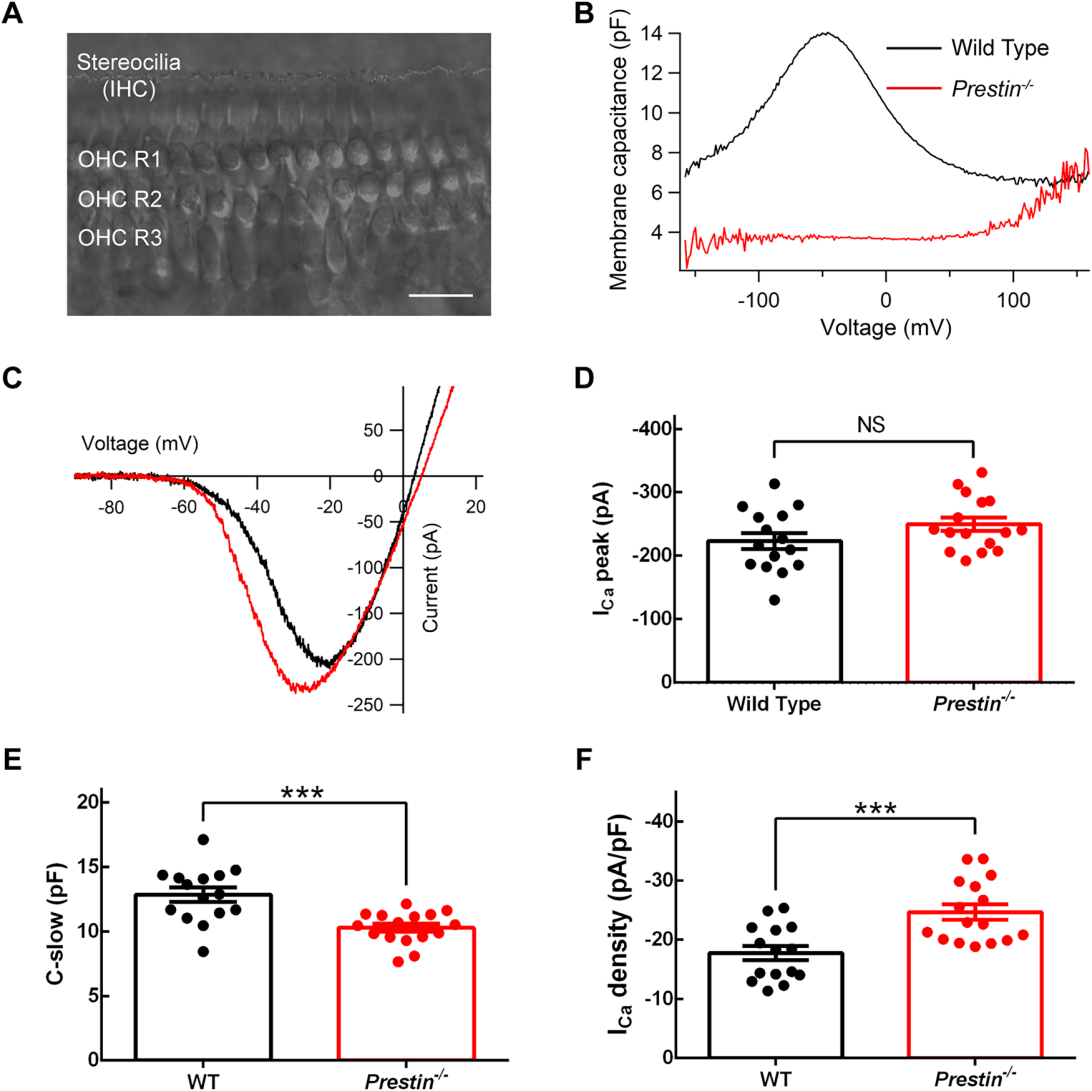
Nonlinear capacitance in OHCs was completely eliminated while calcium current (*I*_Ca_) in IHCs remained unchanged in *Prestin^−/−^* cochleae. ***A***, DIC image of a whole-mount cochlea at the apical turn, showing three rows of OHCs, and one row of stereocilia for IHCs. Scale bar, 20 μm. ***B***, Nonlinear capacitance (NLC) recordings from a pair of WT and *Prestin^−/−^* OHCs, showing that NLC in the *Prestin^−/−^* OHC was completely eliminated. ***C***, Representative *I*–*V* curves for *I*_Ca_ in WT and *Prestin^−/−^* IHCs, showing that *I*_Ca_ was largely intact. ***D–F***, Pooled results for *I*_Ca_ peak (***D***), C-slow (***E***), and *I*_Ca_ density (***F***). WT, *n* = 15 IHCs, *N* = 10 animals; *Prestin^−/−^*, *n* = 16, *N* = 7. C-slow is the capacitance compensated in the patch-clamp amplifier to minimize the capacitive current in response to test pulse, and it is often used as an estimate of whole-cell capacitance. While the *I*_Ca_ peak remained unchanged, the whole-cell capacitance (C-slow) was reduced, yielding a higher *I*_Ca_ density in *Prestin^−/−^* IHCs.

The removal of cochlear amplification is expected to reduce mechanical stimulation received by IHCs, and we therefore wanted to find out if this reduced mechanical stimulation would bear effect on functions of ribbon synapses in IHCs. We first performed patch-clamp recording in OHCs in whole-mount cochleae (Fig. 2*A*), and we found that the OHCs in *Prestin^−/−^* cochleae had a significantly smaller whole-cell capacitance and that the nonlinear capacitance (NLC) in these OHCs was completely eliminated (Fig. 2*B*). We then performed patch-clamp recording in IHCs, applied a voltage ramp, and recorded Ca^2+^ current (*I*_Ca_; Fig. 2*C*). We found that the *I*_Ca_ peak was not significantly changed (224 ± 12.8 pA for WT IHCs, 250 ± 10.5 pA for *Prestin^−/−^* IHCs, two-tailed unpaired *t* test, *p* = 0.115; Fig. 2*D*). The seemingly left shift of the Ca^2+^ current activation in *Prestin^−/−^* IHCs is likely caused by incomplete blockade of K^+^ currents, as it disappeared with stronger K^+^ current blockade (data not shown). Like OHCs, IHCs in *Prestin^−/−^* cochleae also had a significantly smaller whole-cell capacitance (12.9 ± 0.550 pF for WT IHCs, 10.3 ± 0.312 pF for *Prestin^−/−^* IHCs, two-tailed unpaired *t* test, *p* = 0.0003; Fig. 2*E*), which makes the current density for *I*_Ca_ significantly larger in *Prestin^−/−^* IHCs (17.8 ± 1.21 pA/pF for WT IHCs, 24.7 ± 1.32 pA/pF for *Prestin^−/−^* IHCs, two-tailed unpaired *t* test, *p* = 0.0006; Fig. 2*F*).

**Figure 3.**
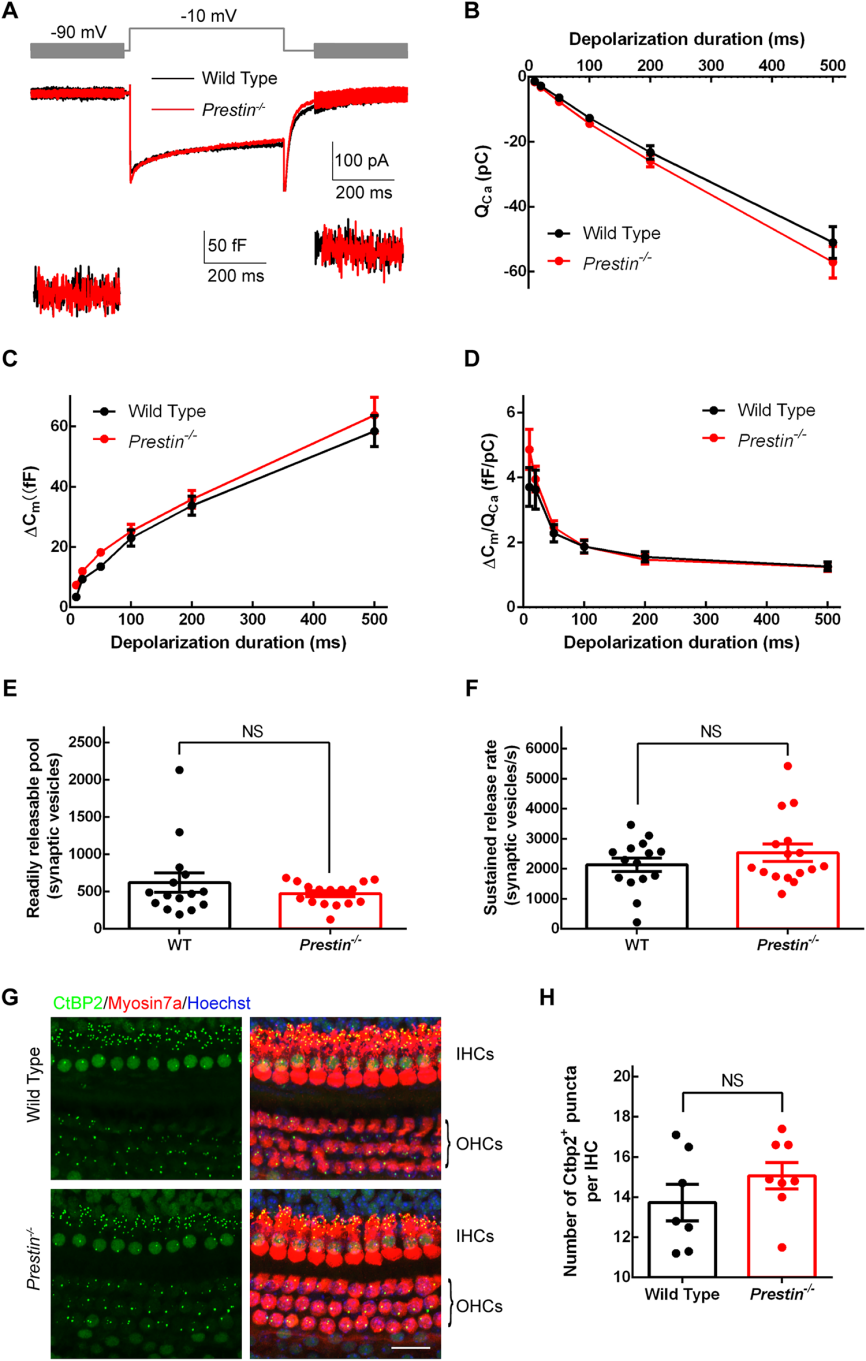
Exocytosis in IHCs was largely intact in *Prestin^−/−^* cochleae. ***A***, Representative *I*_Ca_ and whole-cell capacitance measurement in WT and *Prestin^−/−^* IHCs. ***B–D***, Pooled results for calcium influx (*Q*_Ca_, ***B***), exocytosis (Δ*C*_m_, ***C***), and the ratio Δ*C*_m_/Q_Ca_ (***D***), showing that exocytosis in IHCs was largely intact (WT, *n* = 15 IHCs, *N* = 10 animals; *Prestin^−/−^*, *n* = 16, *N* = 7). ***E***, ***F***, Neither the readily releasable pool (RRP, ***E***) of synaptic vesicles nor the sustained release rate (SRR, ***F***) was significantly changed in *Prestin^−/−^* IHCs (WT, *n* = 15, *N* = 10; *Prestin^−/−^*, *n* = 16, *N* = 7). RRP and SRR are determined from data in ***C*** through curve fitting (see the Materials and Methods for details). ***G***, ***H***, Images of whole-mount WT and *Prestin^−/−^* cochleae, triple immunolabeled for CtBP2, Myosin7a, and Hoechst (***G***) and pooled results for synaptic ribbon counts, showing that the number of synaptic ribbons per IHC was slightly increased in *Prestin^−/−^* IHCs but the difference is not statistically significant (WT, *n* = 7 areas, *N* = 3 animals; *Prestin^−/−^*, *n* = 8, *N* = 3). Scale bar, 20 μm.

We next performed whole-cell capacitance measurement to assess exocytosis from IHCs. As shown in Figure 3*A*, in response to step depolarization of 500 ms, IHCs produced an *I*_Ca_ and a capacitance jump (Δ*C_m_*). We integrated *I*_Ca_ to obtain the Ca^2+^ charge (*Q*_Ca_), and we found no significant difference in *Q*_Ca_ with different stimulation lengths between WT and *Prestin^−/−^* IHCs (Fig. 3*B*), consistent with our finding on the *I*_Ca_ peak described above. Importantly, we found no significant difference in Δ*C_m_*, either (Fig. 3*C*). In a separate set of experiments, we also examined exocytosis induced by stimulation of different depolarization levels, and as expected, we found no significant difference in either *Q*_Ca_ or Δ*C_m_* between WT and *Prestin^−/−^* IHCs (data not shown). Furthermore, we calculated the ratio of Δ*C_m_*/*Q*_Ca_, an indicator for Ca^2+^ efficiency in trigging exocytosis, and once again we found no significant difference between WT and *Prestin^−/−^* IHCs (Fig. 3*D*). To examine kinetics of exocytosis, we performed curve fitting (see the equation in the Materials and Methods) on data in individual IHCs in Figure 3*C*, yielding an estimate of the readily releasable pool (RRP) of synaptic vesicles and the sustained release rate (SRR) of synaptic vesicles, neither of which was significantly altered in *Prestin^−/−^* IHCs (RRP: 621 ± 130 synaptic vesicles for WT IHCs, 472 ± 39.0 synaptic vesicles for *Prestin^−/−^* IHCs, two-tailed unpaired *t* test, *p* > 0.999; SRR: 2133 ± 221 synaptic vesicles/s for WT IHCs, 2,534 ± 285 synaptic vesicles/s for *Prestin^−/−^* IHCs, two-tailed Mann–Whitney U test, *p* = 0.674; Fig. 3*E*,*F*). Lastly, we performed immunolabeling on the apical turn of whole-mount cochleae (Fig. 3*G*), counted the number of synaptic ribbons in IHCs, and found no significant difference (13.7 ± 0.908 for WT IHCs, 15.1 ± 0.658 for *Prestin^−/−^* IHCs, two-tailed unpaired *t* test, *p* = 0.248; Fig. 3*H*). Taken together, these results suggest ribbon synapses in IHCs are largely intact in the absence of cochlear amplification.

### Excitability of bushy cells in the cochlear nucleus was significantly increased in *Prestin^−/−^* mice

**Figure 4.**
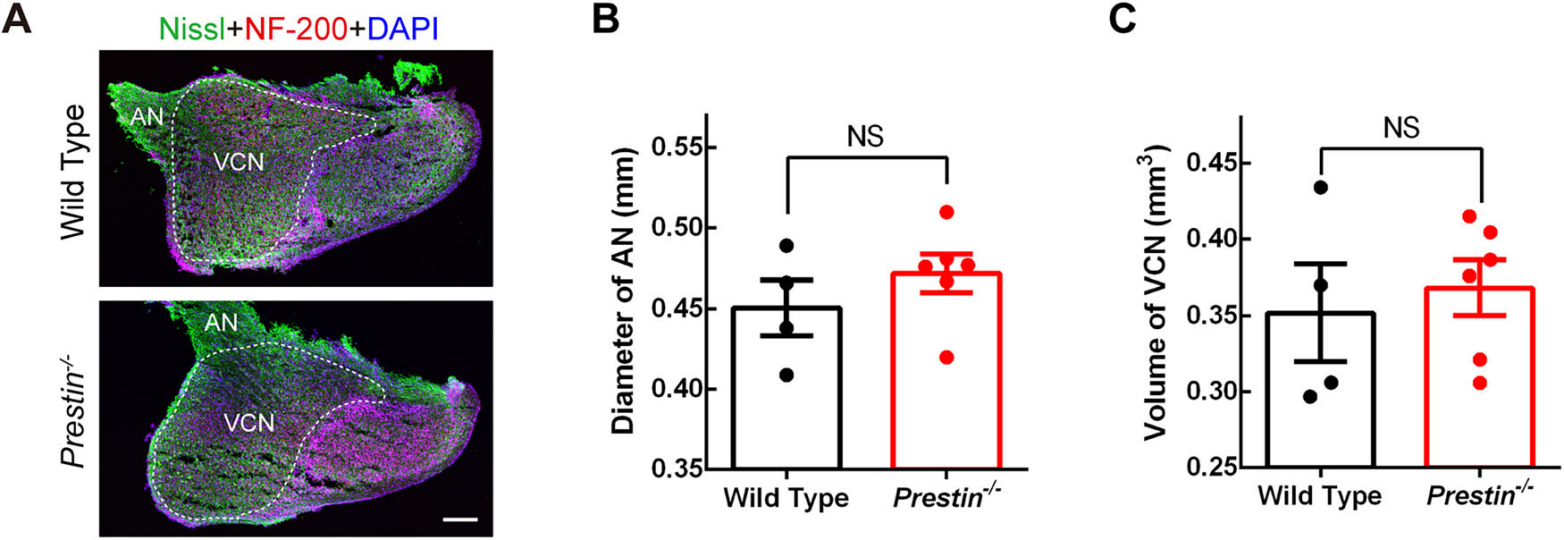
Gross anatomy of the auditory nerve and the cochlear nucleus were intact in *Prestin^−/−^* mice. ***A***, Representative images of fixed sagittal brainstem sections (40 μm thick) in WT and *Prestin^−/−^* mice. The ventral cochlear nucleus (VCN) was outlined with dashed line. ***B***, ***C***, Summary of the auditory nerve stump diameter (***B***) and the VCN volume (***C***), showing neither was significantly altered in *Prestin^−/−^* mice when compared with WT mice (WT, *N* = 4 animals; *Prestin^−/−^*, *N* = 6).

In the central end, auditory nerve fibers (ANFs) make giant synaptic contacts with bushy cells in the cochlear nucleus in the brainstem, which is referred to as the endbulb of Held synapse. In *Otof^−/−^* mice which are born deaf because IHCs are unable to release synaptic vesicles, the cochlear nucleus is significantly smaller, likely due to the lack of spiking on ANFs that drives the development and maturation of auditory pathways (Wright et al., 2014). We wanted to find out if this is the case for *Prestin^−/−^* mice. We therefore collected serial sagittal sections of the cochlear nucleus and performed Nissl and NF-200 staining (Fig. 4*A*). We first measured the diameter of the auditory nerve stump, and we found no significant difference (0.451 ± 0.0173 mm for WT mice, 0.472 ± 0.0120 mm for *Prestin^−/−^* mice, two-tailed Mann–Whitney *U* test, *p* = 0.333; Fig. 4*B*). We next calculated the volume of the ventral cochlear nucleus (VCN) and once again found no significant difference (0.352 ± 0.0319 mm^3^ for WT mice, 0.368 ± 0.0183 mm^3^ for *Prestin^−/−^* mice, two-tailed Mann–Whitney *U* test, *p* = 0.562; Fig. 4*C*). Taken together, these results suggest that in the absence of cochlear amplification, although spiking in ANFs is likely to be reduced in a substantial manner, the remaining spiking is still sufficient to drive normal development of the cochlear nucleus.

**Figure 5.**
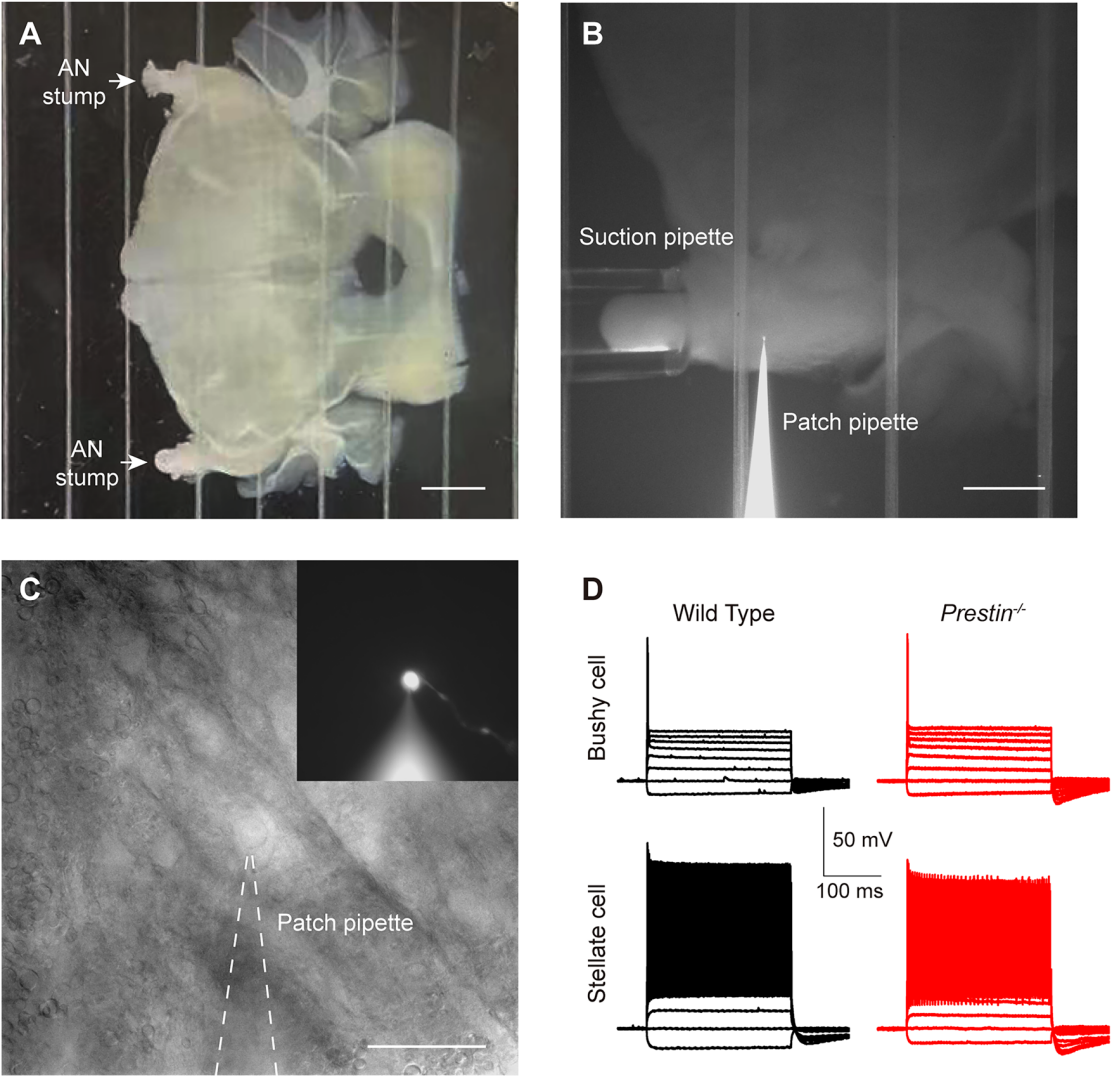
Patch-clamp recording in bushy cells in the cochlear nucleus. ***A***, Image of a coronal brainstem slice under dissection scope, prepared from a *Prestin^−/−^* mouse. Scale bar, 1 mm. ***B***, Image of the stimulation and recording sites under 4× objective in a *Prestin^−/−^* brainstem slice. On the bottom, a patch pipette filled with internal solution containing Alexa 488 was attached to the cell under patch-clamp recording. On the left, a suction pipette was attached the auditory nerve stump, through which brief voltage pulses were applied to stimulate auditory nerve fibers. Scale bar, 500 μm. ***C***, Bright-field and fluorescent (inset) images of the cell from ***B*** under a 60× objective, from the same brainstem slice as in ***B***. In the fluorescent image, a long axon can be seen attached to the soma. Scale bar, 50 μm. ***D***, Representative voltage responses to step current injections for bushy cells (top) and stellate cells (bottom) from WT (left) and *Prestin^−/−^* (right) mice.

**Figure 6.**
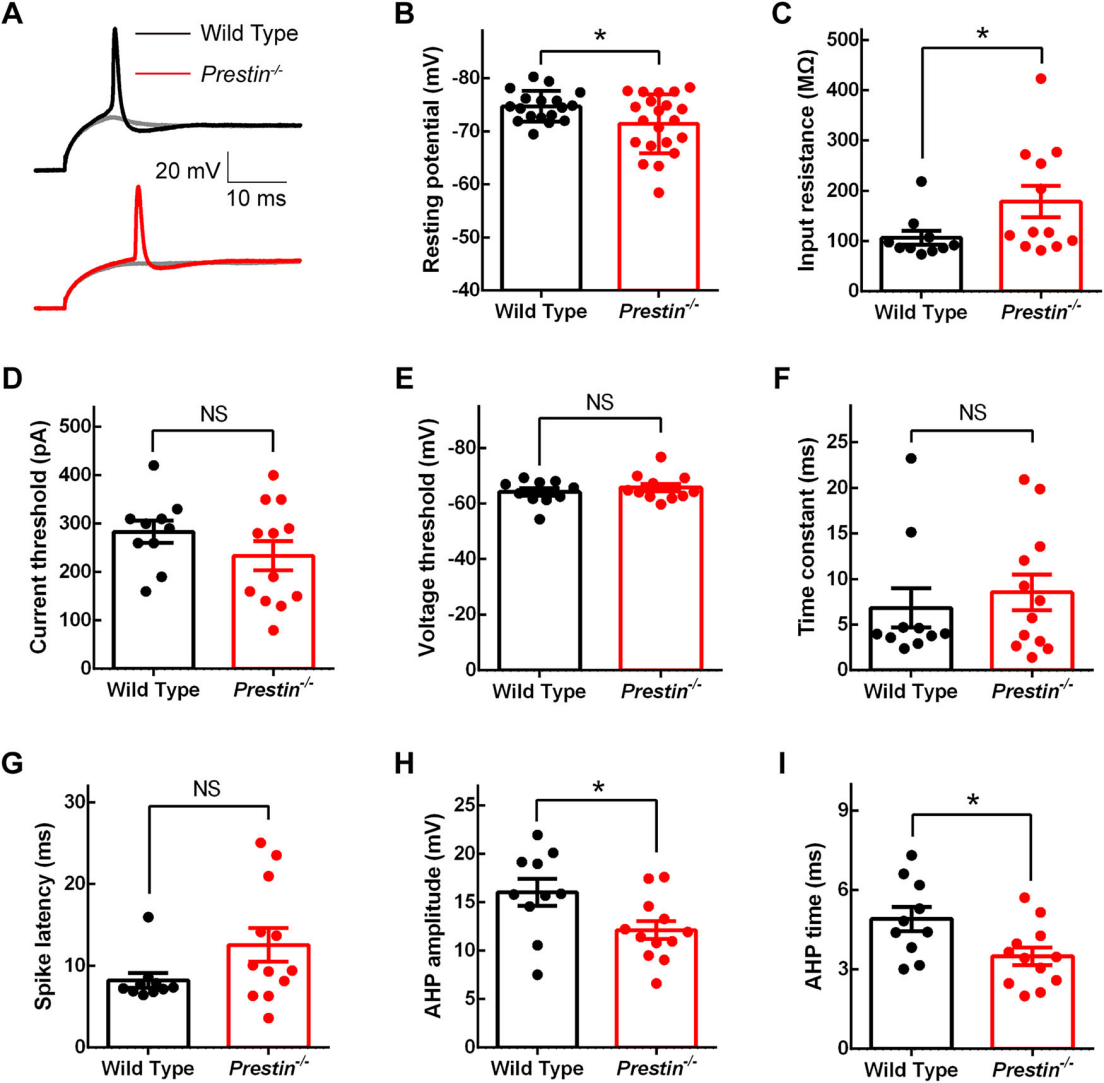
Excitability of bushy cells in the cochlear nucleus of *Prestin^−/−^* mice was significantly increased. ***A***, Representative voltage responses to step current injections for WT and *Prestin^−/−^* bushy cells. For each cell, a voltage response with a spike that required the least amount of current injection was shown, and the current threshold was determined as the corresponding current. Meanwhile, a voltage response with the maximum current injection but without trigging a spike was also shown (in gray), which was used to determine input resistance. ***B–I***, Summary of intrinsic properties, showing that bushy cells in the cochlear nucleus of *Prestin^−/−^* mice had a more depolarized resting membrane potential (***B***), an increased input resistance (***C***), and a smaller and quicker after hyperpolarization (***H***, ***I***), all of which indicate increased excitability. Meanwhile, no difference was found in the current threshold (***D***), the voltage threshold (***E***), the time constant (***F***), or the spike latency (***G***) between WT and *Prestin^−/−^* bushy cells. For data in ***B***, WT, *n* = 18 bushy cells, *N* = 12 animals; *Prestin^−/−^*, *n* = 21, *N* = 12. For data in ***C–I***, WT, *n* = 10, *N* = 8; *Prestin^−/−^*, *n* = 12, *N* = 8.

We next prepared acute brainstem slices and performed patch-clamp recording in bushy cells in anteroventral cochlear nucleus (Fig. 5). We first held the cell at zero current under current-clamp, and we found bushy cells in *Prestin^−/−^* mice had a slightly more depolarized resting membrane potential (−74.7 ± 0.676 mV for WT mice, −71.5 ± 1.21 mV for *Prestin^−/−^* mice, two-tailed unpaired *t* test, *p* = 0.0299; Fig. 6*B*), suggesting increased excitability in bushy cells in *Prestin^−/−^* mice. We next held the cell at a negative current to maintain the membrane potential at −100 mV, then applied step current injection of increasing amplitudes, and we found that bushy cells always fired a single spike (Figs. 5*D*, 6*A*), consistent with their physiological characteristics published by multiple groups (Wu and Oertel, 1984; Xie, 2016; Antunes et al., 2020). We took the voltage response with the largest current injection but without triggering a spike and calculated the input resistance, and we found that it was significantly increased from 107 ± 13.6 MΩ in WT mice to 178 ± 31.1 MΩ in *Prestin^−/−^* mice (two-tailed Mann–Whitney *U* test, *p* = 0.0356; Fig. 6*C*), once again suggesting increased excitability in bushy cells in *Prestin^−/−^* mice. Lastly, we took the voltage response with the least current injection but triggering a spike and calculated parameters of spikes. We found no significant change in the current threshold, the voltage threshold, the time constant, or the spike latency (Fig. 6*D–G*). However, we found that the afterhyperpolarization was significantly smaller (16.0 ± 1.39 mV for WT mice, 12.1 ± 0.937 mV for *Prestin^−/−^* mice, two-tailed unpaired *t* test, *p* = 0.0267; Fig. 6*H*) and briefer (4.91 ± 0.457 ms for WT mice, 3.50 ± 0.334 ms for *Prestin^−/−^* mice, two-tailed unpaired *t* test, *p* = 0.0192; Fig. 6*I*) in *Prestin^−/−^* mice, indicating increased excitability in bushy cells of *Prestin^−/−^* mice. Taken together, we found that the excitability of bushy cells is significantly increased in the absence of cochlear amplification.

### Transmission at the endbulb of Held synapse was greatly altered in *Prestin^−/−^* mice

**Figure 7.**
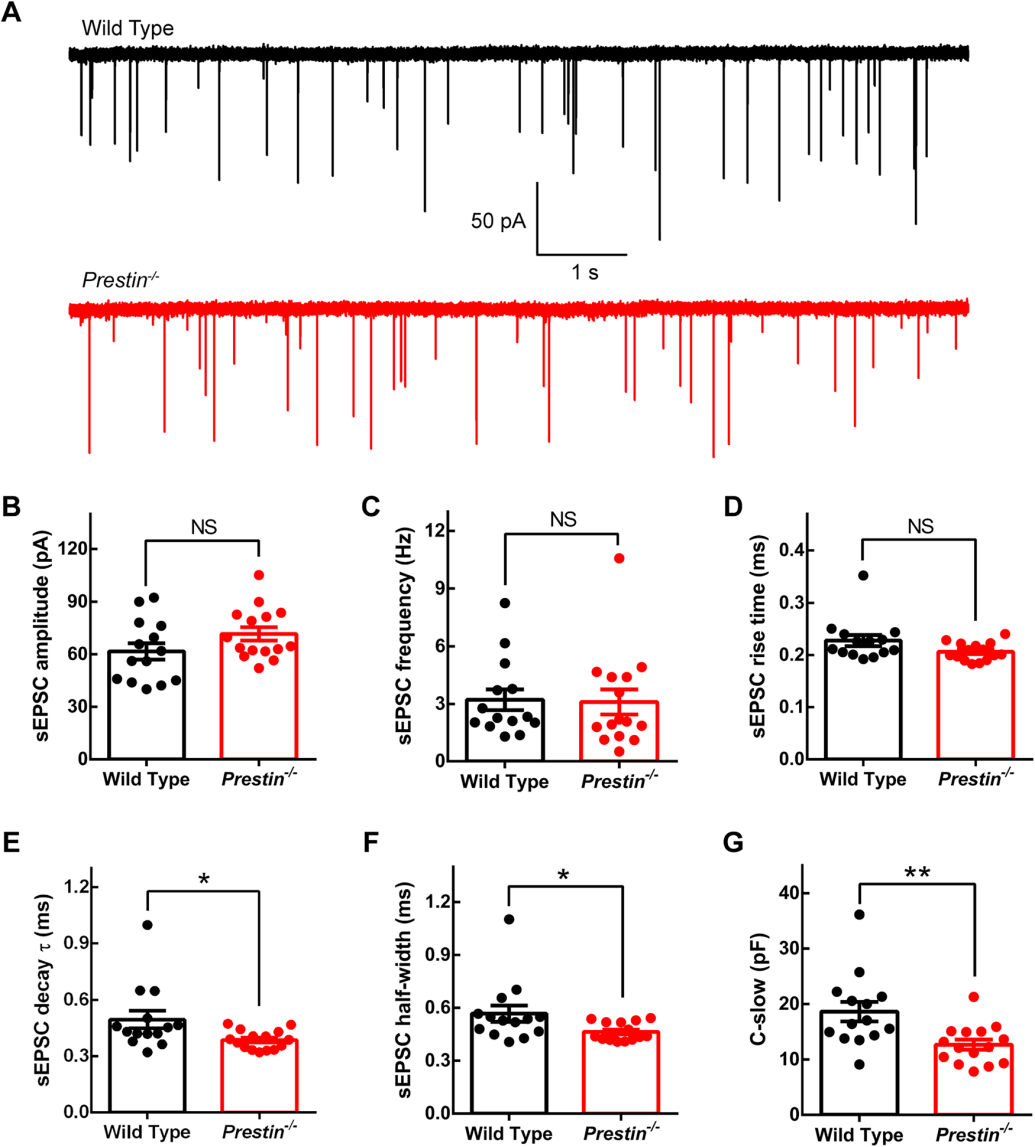
Spontaneous excitatory postsynaptic currents (EPSCs) in WT and *Prestin^−/−^* bushy cells. ***A***, Representative spontaneous EPSC recording in bush cells from WT and *Prestin^−/−^* mice. ***B–G***, Summary of spontaneous EPSCs. Neither the amplitude (***B***) nor the frequency (***C***) of spontaneous EPSCs was significantly changed in *Prestin^−/−^* bushy cells. The rise time was not changed either (***D***), but the decay was significantly briefer for *Prestin^−/−^* bushy cells (***E***), making the half-width briefer in these cells (***F***). Lastly, the whole-cell capacitance (C-slow) became significantly smaller in *Prestin^−/−^* bushy cells (***G***). WT, *n* = 14 bushy cells, *N* = 10 animals; *Prestin^−/−^*, *n* = 15, *N* = 9.

In order to examine if and how the endbulb of Held synapse is changed on the presynaptic side in absence of cochlear amplification, we first recorded spontaneous excitatory postsynaptic currents (EPSCs) in bushy cells of WT and *Prestin^−/−^* mice (Fig. 7*A*). In this slice preparation, ANFs are severed from their soma in the cochlea so that they do not fire spikes spontaneously; therefore, spontaneous EPSCs represent quantal responses. Neither the amplitude nor the frequency of spontaneous EPSCs was significantly changed (Fig. 7*B*,*C*), suggesting that the quantal size of this synapse remains unchanged in absence of cochlear amplification. The rise time was not significantly changed, either (Fig. 7*D*), but the decay time constant was significantly reduced from 0.495 ± 0.0461 ms in WT bushy cells to 0.387 ± 0.130 ms in *Prestin^−/−^* bushy cells (two-tailed Mann–Whitney *U* test, *p* = 0.0222; Fig. 7*E*). Consistently, the half-width was also significantly reduced (0.567 ± 0.0466 ms for WT bushy cells, 0.463 ± 0.0132 ms for *Prestin^−/−^* bushy cells, two-tailed Mann–Whitney *U* test, *p* = 0.0174; Fig. 7*F*). In addition, we found a significantly smaller whole-cell capacitance in *Prestin^−/−^* bushy cells (18.6 ± 1.77 pF for WT bushy cells, 12.7 ± 0.908 pF for *Prestin^−/−^* bushy cells, two-tailed Mann–Whitney *U* test, *p* = 0.0033; Fig. 7*G*), which is consistently observed in mice with reduced cochlear input (Xie, 2016; Connelly et al., 2017).

**Figure 8.**
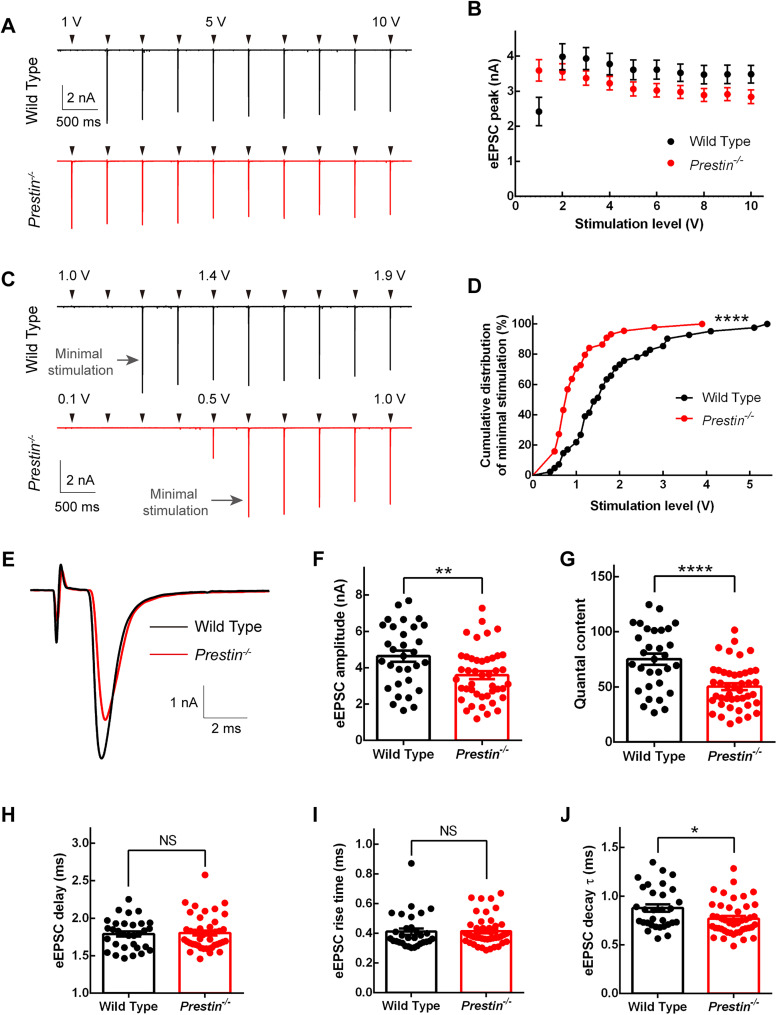
Evoked EPSCs in bushy cells by stimulation of auditory nerve fibers. ***A***, Representative EPSC recordings while auditory nerve fibers were stimulated with increasing voltage levels with an increment of 1.0 V. ***B***, Amplitude of evoked EPSC plotted against voltage level of stimulation (WT, *n* = 46 bushy cells, *N* = 20 animals; *Prestin^−/−^*, *n* = 64, *N* = 23). ***C***, The same as Figure 8*A* but with stimulation increment of 0.1 V. For clarity, the stimulation artifacts are removed, and arrowheads are placed above the traces to indicate timing of stimulation, for both ***A*** and ***C***. ***D***, Cumulative distributions of the minimal stimulation voltage for all cells tested (WT, *n* = 41, *N* = 19; *Prestin^−/−^*, *n* = 44, *N* = 22). The left shift of the distribution for *Prestin^−/−^* mice suggests that their auditory nerve fibers are more excitable to fire spikes. ***E***, Typical evoked EPSCs recorded from WT and *Prestin^−/−^* bushy cells. Note that the stimulation artifact is well separated from EPSC. ***F–J***, Summary properties of evoked EPSCs (WT, *n* = 31, *N* = 17; *Prestin^−/−^*, *n* = 44, *N* = 23). Both the amplitude (***F***) and the quantal content (***G***) were significantly reduced in *Prestin^−/−^* bushy cells. No significant change was found in the synaptic delay (***H***) or the rise time (***I***), but the decay (***H***) became briefer in *Prestin^−/−^* bushy cells.

We next applied a suction pipette on the auditor nerve stump (Fig. 5*B*) and stimulated ANFs extracellularly with brief voltage pulses of increasing amplitude. We first used voltage pulses with an increment of 1 V (Fig. 8*A*), and we found that in WT mice voltage pulses of 1 V either failed to evoke an EPSC or succeeded in evoking an EPSC but with a significantly smaller amplitude compared with that evoked by voltage pulses of 2 V (Fig. 8*B*). For *Prestin^−/−^* mice, however, voltage pulses of 1 V were always sufficient to evoke an EPSC, and the EPSC amplitude is comparable to that evoked by voltage pulses of 2 V, suggesting that ANFs in *Prestin^−/−^* mice are more excitable to fire spikes. We then switched to an increment of 0.1 V, allowing us to determine precisely the minimal stimulation to evoke a large EPSC, which was 1.2 and 0.6 V, respectively, for a pair of WT and *Prestin^−/−^* examples shown in Figure 8*C*. We plotted the minimal stimulation of all cells we tested in WT or *Prestin^−/−^* mice in cumulative distribution, and we found the curve was significantly shifted to the left for *Prestin^−/−^* mice (log-rank test, *p* < 0.0001; Fig. 8*D*), once again indicating elevated excitability in ANFs of *Prestin^−/−^* mice.

To evoke EPSCs with a consistent amplitude in bushy cells, we used voltage pulses with slightly larger amplitude than the minimal stimulation, for all subsequent experiments below. With these stimulations, we found the amplitude of evoked EPSC was significantly reduced from 4.63 ± 0.312 nA for WT mice to 3.61 ± 0.218 nA for *Prestin^−/−^* mice (two-tailed unpaired *t* test, *p* = 0.0067; Fig. 8*E*,*F*), suggesting that the synaptic strength was substantially decreased. By dividing the amplitude of evoked EPSC with the amplitude of spontaneous EPSCs, we obtained the quantal content, which was significantly reduced from 75.1 ± 5.06 for WT mice to 50.4 ± 3.04 for *Prestin^−/−^* mice (two-tailed unpaired *t* test, *p* < 0.0001; Fig. 8*G*). We next divided the quantal content with RRP (see Results; Fig. 10*C*), and we found no significant change in the release probability (0.219 ± 0.0130 for WT mice, *n* = 31 bushy cells, *N* = 17 animals; 0.211 ± 0.0123 for *Prestin^−/−^* mice, *n* = 44, *N* = 23; two-tailed Mann–Whitney *U* test, *p* = 0.305, data not shown), suggesting that the decreased synaptic strength in *Prestin^−/−^* mice is caused solely by reduced RRP. Furthermore, we found no significant difference in the synaptic delay or the rise time between WT and *Prestin^−/−^* mice (Fig. 8*H*,*I*), but the decay time constant was significantly reduced in *Prestin^−/−^* mice (0.878 ± 0.0385 ms for WT mice, 0.770 ± 0.0260 ms for *Prestin^−/−^* mice, two-tailed Mann–Whitney *U* test, *p* = 0.0248; Fig. 8*J*), consistent with our finding on the decay of spontaneous EPSCs described above.

**Figure 9.**
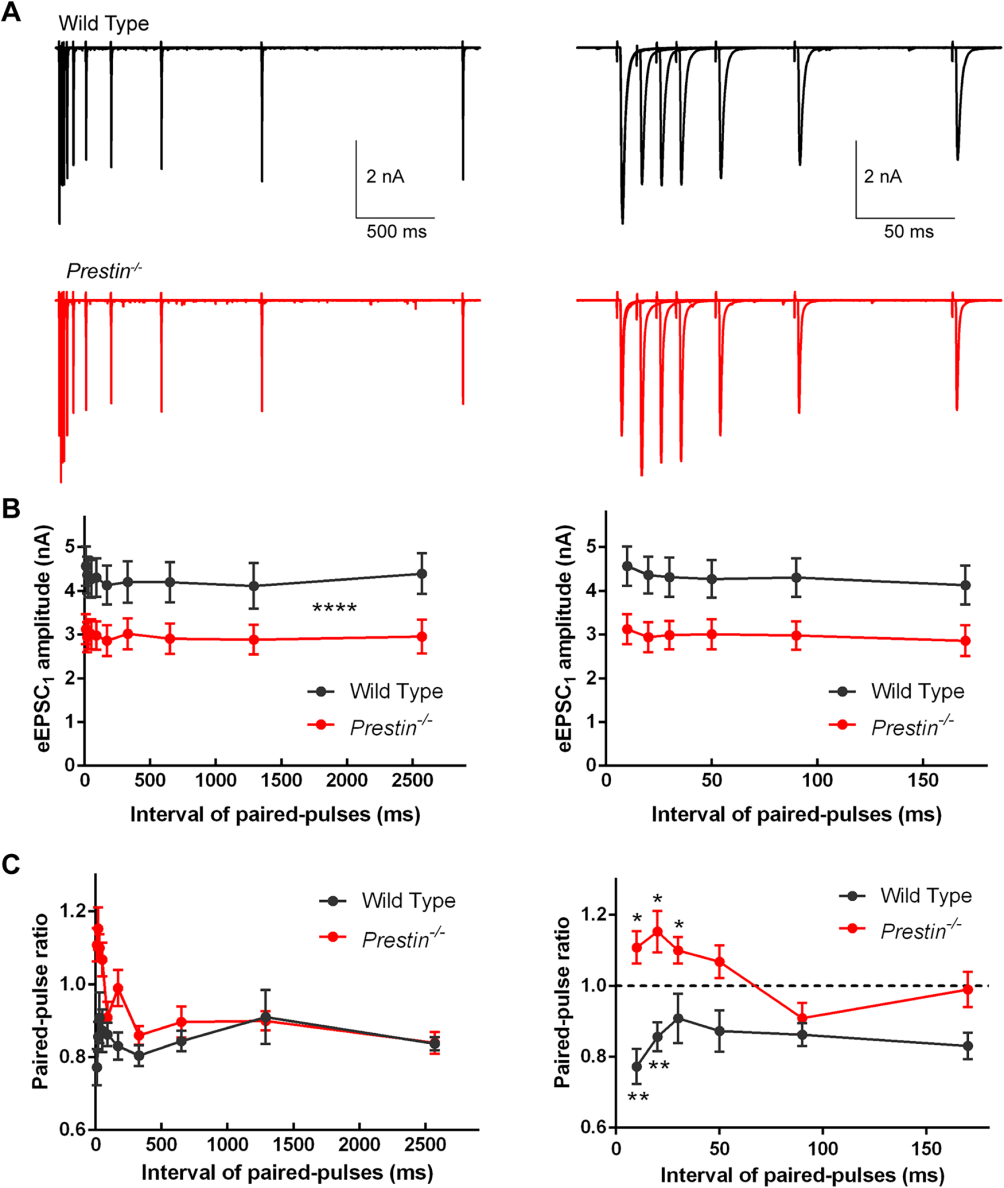
Short-term plasticity in the endbulb of Held synapse was reversed from depression in WT mice to facilitation in *Prestin^−/−^* mice. ***A***, Typical EPSC recordings evoked by paired-pulse stimulations of varied intervals in WT and *Prestin^−/−^* mice. Shown on the right is part of the left with smaller temporal scale. ***B***, Summary of the amplitudes for the first EPSCs, which were significantly decreased in *Prestin^−/−^* mice (WT, *n* = 10 bushy cells, *N* = 9 animals; *Prestin^−/−^*, *n* = 14, *N* = 13). ***C***, Summary of the amplitude ratio EPSC_2_/EPSC_1_, showing that the operation of the synapse switched from depression in WT mice to facilitation in *Prestin^−/−^* mice (WT, *n* = 10, *N* = 9; *Prestin^−/−^*, *n* = 14, *N* = 13).

We then applied stimulation of paired voltage pulses with varied interval and investigated the short-term plasticity at the endbulb of Held synapse (Fig. 9*A*). As shown in Figure 9*B*, the amplitude of the first evoked EPSC was maintained at a steady level over time for both WT and *Prestin^−/−^* mice. Consistent with the finding of reduced amplitude in evoked EPSCs, we found once again the amplitude of the first evoked EPSC was significantly smaller in *Prestin^−/−^* mice (two-tailed Mann–Whitney *U* test, *p* < 0.0001; Fig. 9*B*). We then calculated the paired-pulse ratio across different intervals, and we found significant depression in WT mice (0.773 ± 0.0500 for 10 ms interval, *p* = 0.0039, Wilcoxon signed rank test; Fig. 9*C*), consistent with published studies (Isaacson and Walmsley, 1996; Yang and Xu-Friedman, 2008; Chanda and Xu-Friedman, 2010; Ngodup et al., 2015). In *Prestin^−/−^* mice, however, we found significant facilitation (1.11 ± 0.0456 for 10 ms interval, *p* = 0.0344, one-sample *t* test; Fig. 9*C*), a complete reverse of short-term plasticity from WT mice, indicating a rather dramatic change of synaptic vesicle release in the endbulb of Held synapse after removal of cochlear amplification.

**Figure 10.**
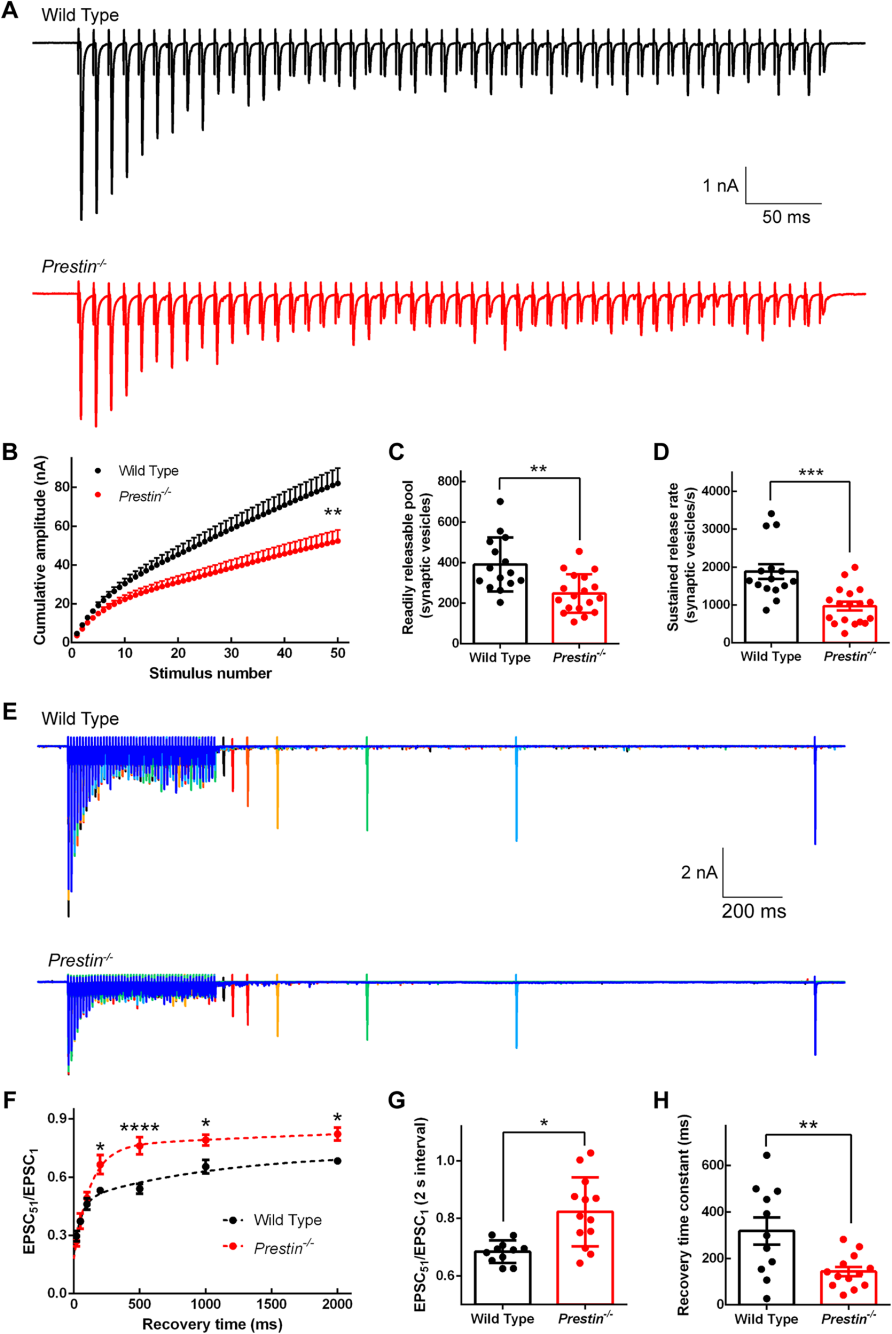
Depletion and refilling of readily releasable pool of synaptic vesicles at the endbulb of Held synapse in WT and *Prestin^−/−^* mice. ***A***, Representative EPSC recordings in response to 50 stimulations delivered at 100 Hz. ***B***, Pooled results for the cumulative EPSC amplitude plotted against the stimulus number, showing that the release of synaptic vesicles was greatly reduced in *Prestin^−/−^* mice (WT, *n* = 15, *N* = 14 animals; *Prestin^−/−^*, *n* = 18, *N* = 13). ***C***, ***D***, pooled results for RRP (***C***) and SRR (***D***), determined from data in ***B*** (see details in the Materials and Methods), both of which were significantly reduced in *Prestin^−/−^* mice (WT, *n* = 15, *N* = 14; *Prestin^−/−^*, *n* = 18, *N* = 13). ***E***, Typical EPSC recordings in response to 50 stimulations at 100 Hz, followed by a 51st stimulation with varied intervals. ***F***, Pooled results for the amplitude ratio EPSC_51_/EPSC_1_ based on data shown in ***E*** from multiple cells (WT, *n* = 11, *N* = 10; *Prestin^−/−^*, *n* = 13, *N* = 9). The dashed lines depict single exponential functions that were fitted to the data. ***G***, ***H***, Refilling of RRP at the endbulb of Held synapse was significantly faster in *Prestin^−/−^* mice, indicated by both a greater recovery ratio at 2 s (***G***) and a shorter recovery time constant (***H***). WT, *n* = 11, *N* = 10; *Prestin^−/−^*, *n* = 13, *N* = 9.

In response to sound stimulation, ANFs fire spikes in expanded time of period at high frequencies, we therefore examined release of synaptic vesicles at the endbulb of Held synapse with 50 stimuli delivered at 100 Hz. In WT mice, we observed synaptic depression as the amplitude of evoked EPSC decreased consecutively and then reached a steady state, an indication of depleting RRP (Fig. 10*A*). The release of synaptic vesicles behaved similarly in *Prestin^−/−^* mice, except that the second evoked EPSC was larger than the first one, consistent with paired-pulse facilitation we found in these mice (see above). We integrated the amplitudes of evoked EPSCs over time, and we found that the release of synaptic vesicles was significantly reduced in *Prestin^−/−^* mice (two-way ANOVA: genotype effect: *F*_(1,31)_ = 8.73, *p* = 0.0059; interval effect: *F*_(49,1519)_ = 178, *p* < 0.0001; interaction, *F*_(49,1519)_ = 11.2, *p* < 0.0001; Fig. 10*B*). Upon further analysis, we determined RRP in WT mice to be 391 ± 34.5 synaptic vesicles, well aligned with previous studies (Butola et al., 2017; Butola et al., 2021). In *Prestin^−/−^* mice, however, RRP was decreased significantly to 248 ± 22.5 synaptic vesicles (two-tailed unpaired *t* test, *p* = 0.0012; Fig. 10*C*). In addition, SRR was also significantly reduced from 1,881 ± 193 synaptic vesicles/s for WT mice to 971 ± 114 synaptic vesicles/s for *Prestin^−/−^* mice (two-tailed unpaired *t* test, *p* = 0.0002; Fig. 10*D*).

In order to probe the refilling of RRP, we applied a 51st stimulus after depleting RRP with 50 stimulations and varied the interval in between (Fig. 10*E*). In Figure 10*F*, we plotted the ratio of EPSC_51_/EPSC_1_ against the interval, and we found that while the initial recovery of RRP was similar between WT and *Prestin^−/−^* mice, the late recovery starting from 200 ms was significantly faster in *Prestin^−/−^* mice (two-way ANOVA: genotype effect: *F*_(1,22)_ = 10.2, *p* = 0.0042; interval effect: *F*_(6,132)_ = 85.4, *p* < 0.0001; interaction: *F*_(6,132)_ = 5.43, *p* < 0.0001; 200 ms interval: *p* = 0.0276, 500 ms interval: *p* < 0.0001, 1,000 ms interval: *p* = 0.0218; 2,000 ms interval: *p* = 0.0193). The ratios of EPSC_51_/EPSC_1_ obtained from individual cells at 2 s interval were redrawn in Figure 10*G*. We then fitted the recovery of RRP to single exponential function, and we found the time constant was significantly reduced from 318 ± 58.1 ms for WT mice to 144 ± 19.9 ms for *Prestin^−/−^* mice (two-tailed unpaired *t* test, *p* = 0.0061; Fig. 10*H*). This faster refilling of RRP in *Prestin^−/−^* mice is surprising, given the reduced sustained release rate after RRP was depleted (Fig. 10*D*). We therefore hypothesized that this faster refilling of RRP in *Prestin^−/−^* mice is likely caused by reduction in the size of RRP. We tested this hypothesis by normalizing the quantal contents of the 51st EPSCs from both animal groups to the RRP of WT mice; we found no significant difference between the recovery of synaptic vesicle release (data not shown), suggesting that the faster refilling of RRP in *Prestin^−/−^* mice is primarily caused by reduction in the size of RRP. Taken together, these results suggest the refilling of RRP becomes quicker at the endbulb of Held synapse in *Prestin^−/−^* mice, indicating that transmission at this first central synapse for hearing is significantly altered in the absence of cochlear amplification.

## Discussion

The emergence of OHCs and cochlear amplification in the mammalian cochlea changes spiking patterns in auditory nerve fibers significantly, but if and how central circuits adapt to this substantial change is poorly understood. In the present study, we took advantage of a *Prestin^−/−^* mouse line to remove cochlear amplification and examined functional changes in both IHC ribbon synapses in the cochlea and the endbulb of Held synapse in the cochlear nucleus. We found that while exocytosis from IHCs in the peripheral was largely intact, transmission at the first central synapse for hearing was greatly altered in absence of cochlear amplification.

### Relative stable transmission in IHC ribbon synapses

In the cochlea of *Prestin^−/−^* mice, although both IHCs and OHCs were significantly smaller in size, as indicated by reduced whole-cell capacitance (Fig. 2*B,E*), we found no significant change in the cellular structure of the basilar membrane (Fig. 1*A*), consistent with a previous report (Liberman et al., 2002). Furthermore, we found no significant change in the Ca^2+^ current (*I*_Ca_) in *Prestin^−/−^* IHCs, either (Fig. 2*D*). Combining with the reduced cell size, we found an increased current density for *I*_Ca_ in *Prestin^−/−^* IHCs (Fig. 2*F*). However, the count of ribbon synapses in IHCs remained unchanged (Fig. 3*G*,*H*); therefore the Ca^2+^ influx through each ribbon synapse is likely to remain the same in *Prestin^−/−^* IHCs. Importantly, we found no significant change in exocytosis in *Prestin^−/−^* IHCs (Fig. 3), indicating that IHC ribbon synapses are largely intact in the absence of cochlear amplification. Similarly, while hyperpolarizing IHCs with potassium channel overexpression diminishes the mechanoelectrical transduction current, functions of IHC ribbon synapses remain unchanged, indicating that transmission at IHC ribbon synapses is rather stable (Carlton et al., 2023). However, it is worth noting here that our examination of IHC ribbon synapses is limited to the apical turn of the cochlea and for mice of 3–4 weeks old only. In fact, for mice of 7–9 weeks old, all IHCs and OHCs have gone missing in the basal turn of the *Prestin^−/−^*cochlea (Liberman et al., 2002). Nevertheless, when compared with significantly altered transmission at the endbulb of Held synapse for mice at the same age, our results indicate transmission at IHC ribbon synapses is relatively stable.

### Change of excitability in bushy cells

In the cochlear nucleus of *Prestin^−/−^* mice, we found that bushy cells exhibited more depolarized resting membrane potential, increased input resistance, and smaller and briefer afterhyperpolarization (Fig. 6), all pointing to increased excitability in these neurons. This increased excitability in postsynaptic bushy cells has been reported in multiple studies, in apparent attempt to neutralize the decrease in presynaptic release of synaptic vesicles. In aged mice with declined presynaptic release of synaptic vesicles, more depolarized resting membrane potential and increased input resistance are observed in bushy cells (Xie, 2016). In *Otof^−/−^* mice where presynaptic release of synaptic vesicles is completely gone, while neither the resting membrane potential nor the input resistance is significantly changed in bushy cells, less steeply rising depolarization is required to trigger spikes (Wright et al., 2014), an indication for increased excitability. For other cases, however, excitability of bushy cells can be changed to boost the change in presynaptic release of synaptic vesicles. In mice with occlusion of the ear canal to mimic conductive hearing loss, bushy cells exhibit decreased input resistance and decreased excitability (Zhuang et al., 2017). In mice exposed to nondamaging noise, bushy cells display increased excitability (Ngodup et al., 2015). While directions and mechanisms vary greatly among different cases, these studies and together with the present study all demonstrate that excitability of bushy cells can be readily modulated by auditory input from the cochlea.

### Change of transmission in the endbulb of Held synapse

For conventional synapses in the brain, synaptic strength is defined as the amplitude of EPSCs evoked by single presynaptic spikes and determined by the quantal size (*q*), the release probability (*p_r_*), and the number of release sites, which is usually approximated with RRP. At the endbulb of Held synapse in *Prestin^−/−^* mice, we found no significant change in *q* (Fig. 7) or *p_r_* (see Results), but RRP was greatly reduced (Fig. 10*C*), causing significant decrease in synaptic strength. When compared with other manipulations of auditory input from the cochlea, this decrease in synaptic strength following the removal of cochlear amplification is rather surprising. Exposure to nondamaging noise decreases *p_r_* and increases RRP (Ngodup et al., 2015), while occlusion of the ear canal increases *p_r_* and decreases RRP (Zhuang et al., 2017), but in both cases, synaptic strength remains unchanged. In two extreme examples where mice are born deaf, synaptic strength is even increased. In *Otof^−/−^* mice, the increase of synaptic strength is caused by increase in both *q* and *p_r_* (Wright et al., 2014), while in *dn/dn* mice, the increase of synaptic strength is attributed to increase in p_r_ only as q remains unchanged (Steel and Bock, 1980; Oleskevich and Walmsley, 2002).

Conventional synapses in the brain are plastic in that their synaptic strength is dynamically modulated by presynaptic activity history, in both long and short term. For short term, the change of synaptic strength is determined, to a large extent, by presynaptic Ca^2+^ dynamics, the size of RRP, and its refilling. At the endbulb of Held synapse, we found that the short-term plasticity was reversed from depression in WT mice to facilitation in *Prestin^−/−^* mice. This is unlikely to be caused by changes in presynaptic Ca^2+^ dynamics, for two reasons. Firstly, we found no significant change in the rate of quantal releases in *Prestin^−/−^* mice, suggesting no significant change in internal Ca^2+^ buffering. Secondly, we found no significant change in *p*_r_ for evoked synaptic vesicle releases in *Prestin^−/−^* mice, indicating no significant change in Ca^2+^ channel density or kinetics. Instead, we found that the size of RRP was reduced (Fig. 10*C*) and that its refilling became faster (Fig. 10*F*), both of which are likely to contribute to the dramatic change of short-term plasticity in *Prestin^−/−^* mice. When compared with other manipulations of cochlear input, this paired-pulse facilitation in absence of cochlear amplification is quite unique. In two cases where cochlear input is also reduced, paired-pulse depression is increased (Wright et al., 2014). In one case where cochlear input is enhanced, paired-pulse depression is nevertheless diminished (Ngodup et al., 2015). In summary, we found transmission at the endbulb of Held synapse was significantly changed in absence of cochlear amplification, and the direction and mechanisms of the change represent significant departure from other manipulations of cochlear input, highlighting depth of complexity of this first central synapse being dynamically modulated.

### Clinical relevance and limitations of the study

OHCs and their prestin expression in humans are vulnerable to insults caused by noise and drugs, and increased prestin level in the serum was found not only in patients with sensorineural hearing loss (Emre et al., 2022) but also in patients exposed to cisplatin, a widely used drug for chemotherapy (Generotti et al., 2022). Furthermore, reduced prestin level in the serum has been linked to the aging of hearing (Parker et al., 2022). All these reports indicate altered cochlear amplification is implicated in different types of hearing loss. The present study, together with studies published by other research groups, firmly establishes dynamic interaction between the cochlear and auditory circuits in the brain. This highlights that one should look beyond the cochlea into the central auditory pathways, not only in searching for mechanisms but also in evaluating treatments, for human patients with hearing loss.

While we tried our best to be inclusive and thorough for the present study, our results are limited, and one should be cautious in interpreting our data. Firstly, due to technical difficulties, our analysis in IHC ribbon synapses in the cochlea is limited to the apical turn only and for mice of 3–4 weeks old only, we cannot rule out the possibility that more prominent changes can be found in IHC ribbon synapses for the middle or basal turn or for the cochlea beyond 1 month. Secondly, we aggregated all recordings in brainstem slices together, which gives us a comprehensive dataset, but we might miss the opportunity to reveal differences at different frequencies. Lastly, because we took a germline approach for our *Prestin^−/−^* mouse model, we cannot rule out possible involvement of developmental changes at the endbulb of Held synapse. However, the fact that IHC ribbon synapses were largely intact (Figs. 2, 3), and that not significant shrinkage of the ventral cochlear nucleus was observed (Fig. 4), suggests the impact of development is minimal. Furthermore, while a conditional knock-out approach might be feasible, additional time is needed for the effect to happen, requiring patch-clamp recording in brainstem slices to be performed beyond 1 month, which is notoriously difficult because myelination in the brainstem becomes dense. In summary, we took the best approach available to the field, and our study represents valuable effort to address a longstanding question of significance in hearing.
